# Cutting Edge: Probiotics and Fecal Microbiota Transplantation in Immunomodulation

**DOI:** 10.1155/2019/1603758

**Published:** 2019-04-16

**Authors:** Wenjie Zeng, Jie Shen, Tao Bo, Liangxin Peng, Hongbo Xu, Moussa Ide Nasser, Quan Zhuang, Mingyi Zhao

**Affiliations:** ^1^Transplantation Center of the 3rd Xiangya Hospital, Central South University, Changsha, Hunan 410013, China; ^2^Xiangya School of Medicine, Central South University, Changsha, Hunan 410013, China; ^3^Pediatric Department of the 3rd Xiangya Hospital, Central South University, Changsha, Hunan 410013, China; ^4^Department of Surgery of the 3rd Xiangya Hospital, Central South University, Changsha, Hunan 410013, China; ^5^Guangdong Cardiovascular Institute, Guangdong General Hospital, Guangdong Academy of Medical Sciences, Guangzhou, Guangdong 510100, China; ^6^Research Center of National Health Ministry on Transplantation Medicine, Changsha, Hunan 410013, China

## Abstract

Probiotics are commensal or nonpathogenic microbes that confer beneficial effects on the host through several mechanisms such as competitive exclusion, antibacterial effects, and modulation of immune responses. Some probiotics have been found to regulate immune responses via immune regulatory mechanisms. T regulatory (Treg) cells, T helper cell balances, dendritic cells, macrophages, B cells, and natural killer (NK) cells can be considered as the most determinant dysregulated mediators in immunomodulatory status. Recently, fecal microbiota transplantation (FMT) has been defined as the transfer of distal gut microbial communities from a healthy individual to a patient's intestinal tract to cure some immune disorders (mainly inflammatory bowel diseases). The aim of this review was followed through the recent literature survey on immunomodulatory effects and mechanisms of probiotics and FMT and also efficacy and safety of probiotics and FMT in clinical trials and applications.

## 1. Introduction

Probiotics were defined in 2002 by experts from the Food and Agriculture Organization of the United Nations and the World Health Organization, and the definition was updated by the International Scientific Association in 2013 [1]. The definition states that probiotics are “live strains of strictly selected microorganisms which, when administered in adequate amounts, confer a health benefit on the host.” Probiotic products are commonly known to be microecological preparations and are used to improve the structure of intestinal flora, inhibit the growth of harmful microorganisms, and enhance the immunity of the human body. To be considered microecologics, probiotics must satisfy the following conditions [2]: be live microorganisms; stay alive and stable before use after culture, production, and storage; be resistant to gastric acid, bile, and trypsin, and remain alive to colonize and proliferate in the intestinal tract; be scientifically proven to be beneficial to the host; and be proven to be safe and reliable or an member of the original intestinal microflora. Currently, the extensively studied and developed probiotics include the related bacteria of *Lactobacillus*, *Bifidobacteria, Escherichia coli (E. coli),* and *Enterococcus* and some yeasts [3].

Currently, as a means of intestinal microecological regulation in addition to microecological preparations, fecal microbiota transplantation (FMT) has become popular in recent years. FMT refers to the transplantation of functional bacteria in the feces of healthy donors into the gastrointestinal tract of the patient to restore the balance of the intestinal microecology, which subsequently treats diseases associated with disorders of intestinal microorganisms. As far back as traditional medical treatments in the fourth century of China, there have been relevant records of FMT treatment [4]. In the era of modern medicine, the earliest report of FMT was in 1958. Eiseman et al. successfully used FMT to treat a case with pseudomembranes [5]. The first report of FMT application in the treatment of *Clostridium difficile* (*C. difficile)* infection (CDI) was in 1983 [6]. In 2010, the United States recommended FMT as a treatment plan for CDI in their clinical guidelines [7]. FMT has now been deemed the primary therapy for refractory and relapsed CDI. In recent years, FMT has become a research focus on biomedicine and clinical medicine. FMT has also been clinically applied to inflammatory bowel disease (IBD), irritable bowel syndrome, chronic functional constipation, intestinal cancer, foodborne allergic gastroenteropathy, and so on [8], and researchers have achieved a certain clinical efficacy. Recently, some studies have shown that there is a very strong potential application for FMT in the field of nongastrointestinal diseases, such as treating arteriosclerosis, metabolic syndrome, diabetes, hepatic encephalopathy, neurodegenerative diseases, among others [9].

## 2. Probiotics and the Immune System

Relevant studies on the mechanism of probiotics mainly focus on the intestinal tract. However, the effect of probiotics is not confined to the initial infection site, and probiotics can work throughout the entire body via the immune system. In gut-associated lymphoid tissues (GALT), probiotic and antigen substances from its metabolites are phagocytized or internalized by M cells to form endosomes. Antigens in M cells are rapidly released and taken in by dendritic cells (DCs), which can transport the antigens to local lymph nodes and then activate naive T and B cells to differentiate into different effector subpopulations, initiating the release of the corresponding cytokines and displaying different immune functions.

A number of studies show that the mechanisms of probiotics include (1) enhancement of the chemical and biological barriers in the intestinal tract as well as regulation of the balance of intestinal flora. Through a space-occupying effect, competition, or antagonism [10–14], and by secreting antibacterial or bactericidal substances (e.g., bacteriocin), increasing digestive enzyme activity, producing organic acid, and so on [15], probiotics can exert an antibacterial effect, maintain the function of intestinal epithelial cells, prevent pathogenic bacteria adhesion, and inhibit the growth of pathogenic bacteria. (2) Through increasing the synthesis of tight junction proteins between epithelial cells [16, 17], probiotics stimulate and promote the expression and secretion of mucous glycoproteins [18], enhance the integrity of intestinal epithelial cells, strengthen the mechanical barrier function of the intestinal tract, and prevent the displacement of intestinal bacteria and endotoxins. (3) Probiotics regulate innate and adaptive immunity, including promoting the development and maturation of the immune system [19], enhancing the viability of macrophages and natural killer (NK) cells [20], stimulating the secretion of secretory immunoglobulin A (sIgA) [21], activating related immune responses mediated by Toll-like receptor (TLR) and nucleotide-binding oligomerization domain-containing protein- (NOD-) like receptors (NLR), regulating the T helper cell (Th)1/Th2 immune response, increasing the number of regulatory T cells (Treg) that secrete interleukin- (IL-) 10 and transforming growth factor (TGF)-*β*, and strengthening their function as well as reducing the level of allergen-specific IgE [22].

**Figure 1:**
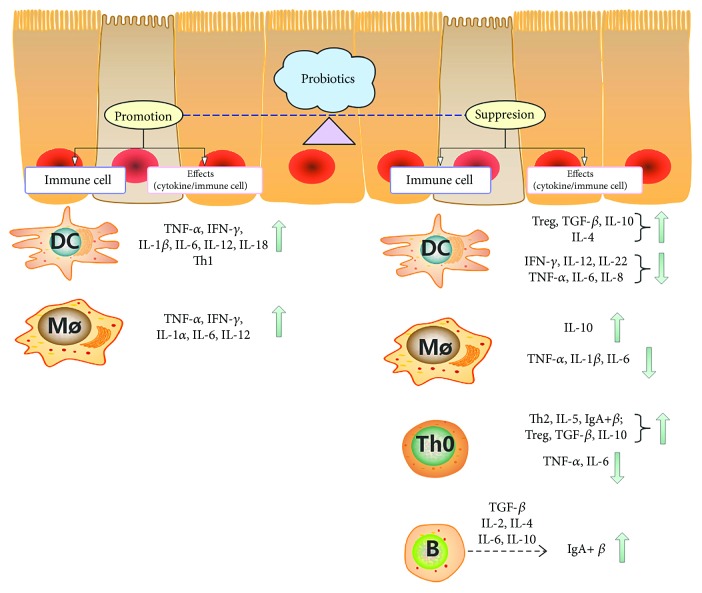
The dual functions of probiotics on the immune system in in vitro and animal experiments. ↑: activity enhanced or quantity increased; ↓: activity reduced or quantity decreased. Immune cell: immune cells on which probiotics directly stimulate. Effects: the immunological effect generated by immune cells stimulated by probiotics, mainly including the regulation on cytokines and the differentiation of related immune cell subpopulations.

The role of probiotics in the immune system is complex. The immune stimulations induced by probiotics are manifested as an increase in the generation of immunoglobulins, enhanced activity of macrophages and lymphocytes, and stimulation of interferon- (IFN-) *γ*. Probiotics that inhibit the immune system are mainly embodied in their anti-inflammatory action.  Figure 1 summarizes the dual function of probiotics in the immune system in in vitro and animal experiments.

Additionally, there is a mechanism behind positive and negative effects of probiotics on the immune system; yet, the exact molecular mechanisms for these commensal-host interactions are poorly described. Many immunomodulatory biologically active signaling molecules of probiotics are microbial-associated molecular patterns (MAMP) that interact with transmembrane host pattern recognition receptors (PRRs). TLR has been the most studied. In addition, extracellular C-type lectin receptors (CLRs) and intracellular NLR can also transmit signals by interaction with bacteria.  Table 1 summarizes the immunomodulatory components of the most common probiotics, *Lactobacillus* and *Bifidobacterium*. However, the molecular basis of these effector-mediated strain-specific probiotics needs to be thoroughly investigated.

Importantly, studies have shown that there are some differences in the physiology and metabolism between probiotic strains from different species and that their effects on the human body are different. Even the functions of different strains from the same species can vary greatly. Similarly, different doses of the same strain can produce different effects. Additionally, there are some differences in function in different hosts. Therefore, the functions of probiotics need to be verified at the strain level to clarify the efficacy of the strain.

## 3. FMT and the Immune System

FMT can increase the microbial diversity of the intestines, maintain the intestinal microecological balance, and rebuild the function of the immune system. Related mechanisms may include (1) intestinal flora introduced from healthy donors that can maintain the intestinal epithelial integrity of patients, limit intestinal permeability, and inhibit intestinal epithelial cell apoptosis to reestablish the function of the intestinal barrier (this may be related to the mechanisms of the intestinal flora from donors that inhibit the adhesion between intestinal pathogens and intestinal epithelial cells (IECs), reduce the damage of IECs, and increase the production and expression of mucosal IgA and mucin by colonizing resistance and producing immunomodulatory molecules and bacteriocin, etc.); (2) the intestinal flora of the donors can also fight against proinflammatory cytokines by directly synthesizing anti-inflammatory factors, reducing local and systemic inflammatory responses; (3) FMT restores the metabolism of secondary bile acids in the intestines, which makes the metabolism of secondary bile acids in the gastrointestinal tract of patients similar to that of donors; (4) competition or antagonism with pathogenic bacteria; and (5) improving insulin resistance. As a result, the patient's immunity is improved [23–27]. Applications in patients confirmed that the effects of FMT on the intestinal microflora of patients are long lasting and mostly safe, with few adverse effects [28]. In addition, FMT can improve anxiety and depression through mechanisms associated with the brain-intestine axis and improve the quality of life of patients [29].

With FMT, the intestinal flora of healthy donors may maintain the microenvironment of recipients and eventually reconstruct the recipient's intestinal ecological balance. The mechanisms can affect the disease processes of gastrointestinal and extraintestinal diseases by altering the mucosal cell gene expression, the intestinal mucosal immune function, the intestinal ecological environment, and body metabolism, which regulate the immune response, the inflammatory response, and the number and activity of neurotransmitters.

## 4. Immunomodulatory Effects and Mechanisms of Probiotics and FMT

### 4.1. Th1/Th2 Balance

Th1 activates macrophages and neutrophils to promote an inflammatory response by secreting IL-2, IL-3, IFN-*γ*, and tumor necrosis factor- (TNF-) *α*. Th2 can secrete IL-4, IL-5, IL-6, IL-10, and IL-13 to activate mast cells and basophils to participate in allergic reactions. Many experiments have shown that probiotics can participate in the negative regulation of the immune system, such as anti-inflammation and antiallergy effects through affecting the Th1/Th2 balance.

#### 4.1.1. Anti-inflammatory Effects

By oral administration of *Lactobacillus plantarum* (*L. plantarum*) A7 and *Bifidobacterium animalis* (*B. animalis*) PTCC 1631 to mice with autoimmune encephalomyelitis (EAE), Salehipour et al. found that naive T cells preferred to differentiate to Th2 cells because of increased production of transcription factor GATA3, which eventually led to the secretion of more IL-4 and IL-10 [30]. Mi et al. found that by orally administering *Bifidobacterium infantis* (*B. infantis*) to colorectal cancer mice induced by dimethylhydrazine, CD4+IL-17+ cells were reduced, resulting in decreased secretion of IL-2, IL-12, and IFN-*γ* from Th1 and Th17. Therefore, *B. infantis* could inhibit intestinal mucositis caused by chemotherapy drugs in colorectal cancer mice [31]. In addition, Rebeca's research showed that after feeding *B. animalis* ssp lactis CNCM-I2494 to low-level inflammatory mice induced by dinitrobenzene sulfonic acid, the number of Th2 cells and the levels of IL-4, IL-5, and IL-10 increased, which significantly improved the barrier permeability diseases [32]. Interestingly, oral administration of *Clostridium butyricum* (*C. butyricum*) CGMCC0313.1 to nonobese diabetic mice resulted in a significant reduction of Th1 and IFN-*γ* secretion in the spleen and an increase of Th2 and IL-4 [33]. Additionally, the serum IgE and IL-4 levels in atopic dermatitis mice were reduced by oral administration of *Lactobacillus casei (L. casei)* variety rhamnosus (LCR35). Moreover, the recovery of the Th1/Th2 balance improves intestinal flora [34]. In the study by Zheng et al., after feeding *Bifidobacterium breve (B. breve*) to colitis mice, the expression levels of IL-4, IL-5, IL-13, and IL-23 message ribonucleic acid (mRNA) in colon tissue increased. In subsequent studies, they also cocultured peripheral blood mononuclear cells (PBMCs) with *B. breve* and found that Th1 and Th17 decreased and Th2 and Treg increased [35].

#### 4.1.2. Antiallergic Effects

In the mouse model of ovalbumin (OVA) allergy, after oral administration of *Lactobacillus bulgaricus* (*L. bulgaricus*), *Streptococcus thermophilus* (*S. thermophilus*), and *Lactobacillus paracasei* (*L. paracasei*) ssp. paracasei CNCMI-1518, the number of Th2 cells and serum IgE decreased but serum IL-10 and IFN-*γ* increased in mice [36]. Similarly, after feeding *Lactobacillus rhamnosus* (*L. rhamnosus*) MTCC 5897 to OVA allergy mice, serum IL-4 decreased, whereas serum IFN-*γ* increased [37]. In a mouse model of whey protein hypersensitivity, oral administration of *Lactobacillus acidophilus* (*L. acidophilus*) and *Bifidobacterium bifidum* (*B. bifidum*) increased the levels of IFN-*γ*, IL-10, and IL-12 and decreased the level of IL-4 in the spleen [38]. Another experiment also showed that differentiation of Th1 increased in mesenteric lymph nodes (MLN) and the spleen and the serum histamine concentration decreased after oral administration of *Bifidobacterium lactis* (*B. lactis*), *L. casei*, *L. rhamnosus*, and *L. plantarum* to mice that were allergic to whey protein [39]. In addition, after feeding *L. plantarum* CJLP133 and CJLP243 to mice with allergic rhinitis caused by birch pollen (BP), the researchers found an increase in IFN-*γ* and decrease in IL-4, IL-5, and IL-13 in bronchoalveolar lavage fluid (BALF). At the same time, serum IL-4, IL-5, IL-13, IgE, and BP-specific IgG1 were also reduced [40].

### 4.2. Th17/Treg Balance

**Figure 2:**
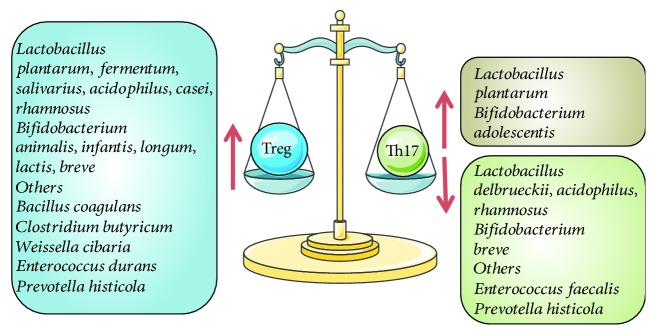
Effects of probiotics on the Th17/Treg balance. Treg can be increased by probiotics, such as *Lactobacillus* (*plantarum*, *fermentum*, *salivarius*, *acidophilus*, *casei*, and *rhamnosus*), *Bifidobacterium* (*animalis*, *infantis*, *longum*, *lactis*, and *breve*), and *Bacillus coagulans*, *Clostridium butyricum*, *Weissella cibaria*, *Enterococcus durans*, and *Prevotella histicola*. Th17 can be increased by probiotics, such as *Lactobacillus plantarum*, *Bifidobacterium adolescentis*. Conversely, Th17 can be decreased by probiotics, such as *Lactobacillus* (*delbrueckii*, *acidophilus*, and *rhamnosus*), *Bifidobacterium breve*, and *Enterococcus faecalis* and *Prevotella histicola*.

Probiotics can affect the Th17/Treg balance in the host immune system. When probiotics promote the differentiation of Th0 to Treg, the clinical effect is to negatively regulate the host immune system. Conversely, when probiotics promote the differentiation of Th0 to Th17, the clinical effect of probiotics is to positively regulate the host immune system. Treg can secrete TGF-*β*, IL-10, and IL-35 to participate in negative immune regulation. Th17 can secrete IL-17, IL-21, and IL-23 to participate in positive immune regulation. To provide a better understanding of this section, we summarized the available literature in Figure 2.

#### 4.2.1. Anti-inflammatory Effects

In experiments with mice that had autoimmune encephalitis (EAE), Ménard et al. found that after feeding *L. plantarum* A7 and *B. animalis* PTCC 1631, the transcription factor Foxp3 of naive T cells increased, resulting in increased Treg differentiation and IL-10 production [41]. In addition, a study by Kwon et al. showed that IRT5 (a mixture of five probiotics) not only increased the levels of Treg and IL-10 in superficial lymph nodes of EAE mice but also reduced the amount of Th17 and secretion of IFN-*γ*, TNF-*α*, and IL-17 [42]. In the study by Mangalam et al., after feeding *Prevotella histicola (P. histicola)* to EAE mice, they found that the amounts of Th1 and Th17 decreased in the MLN and spleen, while the numbers of Treg, regulatory dendritic cells (DCreg), and suppressive macrophages increased [43]. Therefore, what is the possible pathway through which probiotics affect T cells? The study by Haghikia and colleagues provides an answer. They fed propionic acid (a metabolite of probiotic) to EAE mice and found that the JNK1 and p38 pathways in naive T cells were inhibited, leading to increased expression of Foxp3 and IL-10 mRNA as well as the promotion of the differentiation of naive T cells to Treg [44]. In addition, in experiments with colitis mice, Qiu et al. [45], Rodríguez-Nogales et al. [46], and Kanda et al. [47] found that probiotics promoted naive T cell differentiation to Treg and increased IL-10 secretion. Moreover, after giving oral *L. acidophilus* to colitis mice induced by dextran sulfate sodium (DSS), they found that not only Treg and IL-10 were increased but also IL-17 was decreased in the spleen. Additionally, the levels of IL-6, TNF-*β*, IL-1*β*, and IL-17 also decreased in colon tissue [48]. In the same model, Kim et al. found that activation of nuclear factor kappa B (NF-*κ*B) was inhibited and the endoplasmic reticulum (ER) pressure signal pathway was disturbed, leading to increased expression of IL-10 in the colon and increased levels of Th2 and Treg in the spleen [49]. In addition, studies have shown that after oral administration of a mixture of 12 probiotics, zinc, and CoQ10 to arthritic mice induced by collagen, Th17 decreased but Treg increased in the spleen. Moreover, the secretion of TNF-*α*, IL-1*β*, IL-6, and IL-17 decreased in the joint synovium. At the same time, the levels of IgG, IgG1, and IgG2a in the serum were reduced [50]. Cortes-Perez et al. found that after intragastric administration of *L. casei* BL23, the number of Foxp3+ROR*γ*t+T cells (type 3 Treg) increased [51]. By oral administration of *Weissella cibaria (W. cibaria)* WIKIM28 to mice with chronic inflammatory skin disease induced by 2,4-dinitrochlorobenzene, Lim et al. found that serum IgE decreased but Treg and IL-10 increased in MLN [52]. After feeding *L. acidophilus* to mice with ulcerative colitis, Chen et al. found that phosphorylation of STAT3 was inhibited, which subsequently caused increased secretion of IL-17 and TNF-*α* [53]. After feeding *C. butyricum* CGMCC0313.1 to autoimmune nonobese diabetic mice, *α*4*β*7+ Tregs increased in the pancreatic LN. This change restored the intestinal microbial disorders caused by diabetes [33].

#### 4.2.2. Antiallergic Effects

In the OVA-allergic mouse, Kim et al. showed that feeding *L. rhamnosus* (Lcr35) could result in increased Treg but decreased IL-4 and IL-17 in MLN, and the response of thymic stroma lymphocytes was weakened [54]. In addition, studies have shown that oral administration of *Enterococcus faecalis (E. faecalis)* FK-23 to OVA-allergic mice can reduce the number of IL-17-expressing CD4+ cells in the lungs, spleen, and intestine. Additionally, the total number of white blood cells and mast cells decreased in BALF [55]. Fu et al. discovered that after feeding *Bacillus coagulans (B. coagulans)* 09.712 to mice allergic to the prion troponin, the mTOR pathway was inhibited in naive T cells, which caused an increase in Foxp3 expression. Additionally, naive T cells differentiated into Treg, which increased the secretion of IL-10 by Treg and decreased the secretion of IL-17A and IL-6 by Th17 [56]. Furthermore, oral administration of *L. casei* DN-114 001 to allergic dermatitis mice increased the number of Treg in the skin and the levels of IL-10 in LN [57]. Salehipour et al. found that the number of Treg was increased in MLN and the spleen, whereas serum histamine decreased but IL-10 increased after feeding *B. lactis*, *L. casei*, *L. rhamnosus*, *L. plantarum*, and sodium butyrate to mice allergic to whey protein [30]. In the study by Zhang et al., oral administration of *C. butyricum* CGMCC0313-1 increased the number of Treg and decreased the serum IL-4, IL-5, IL-13, and IL-17 levels in mice with an intestinal allergy induced by lactoglobulin [58].

#### 4.2.3. Other Aspects of Negative Immune Regulation

In the study by Laskowska et al., feeding bokashi preparations (a mixture of 11 probiotics) to pregnant sows increased serum IL-10 as well as IL-10 and TGF-*β* in the colostrum [59]. Moreover, in some experiments, probiotics also regulate autoimmune diseases. For example, in systemic lupus erythematosus (SLE) mice induced by pristane, feeding *L. rhamnosus* and *Lactobacillus delbrueckii* (*L. delbrueckii*) reduced the expression of ROR*γ* mRNA, downregulated Th1 and Th17 cells, and decreased the levels of IFN-*γ* and IL-17 [60].

#### 4.2.4. Positive Immune Regulation

When probiotics promote the differentiation of Th0 cells into Th17 cells or inhibit the differentiation of Th0 cells into Treg, they can positively regulate the host immune system. Tan et al. found that feeding *Bifidobacterium adolescentis* (*B. adolescentis*) could increase the number of Th17 in the gut [61]. Xie et al. found that by oral administration of *L. plantarum* NCU116 to immune suppressive mice induced by high-dose cyclophosphamide, the expression of TLR-2 and TLR-6 mRNA increased in the small intestine, which resulted in an increase of Th17 cells and the IL-17, IL-21, IL-23, and TGF-*β*3 levels [62].

### 4.3. B Cells

B cells can differentiate into plasma cells or regulatory B cells (Breg). Plasma cells can synthesize and secrete antibodies and are mainly involved in humoral immunity. Breg can perform immunological negative regulation by producing IL-10 or TGF-*β*. When probiotics promote the differentiation of B cells into plasma cells, positive regulation of the immune system can be achieved. When probiotics promote the differentiation of B cells into Breg, they can negatively regulate the immune system. Shi et al. fed *C. butyricum* to OVA-allergic mice and found an increasing number of IL-10-producing OVA-specific B cells (OVAsBC). Furthermore, they cocultured OVAsBC, OVA, and *C. butyricum* and showed that OVAsBC differentiated towards Breg and the secretion of IL-10 increased [63]. In addition, studies have shown that *Lactobacillus helveticus* (*L. helveticus*) SBT2171 stimulated B cells isolated from mouse spleens, which could inhibit lymphocyte proliferation by inhibiting the JNK signaling pathway [64]. In addition, Sakai et al. showed that after oral application of *Lactobacillus gasseri (L. gasseri)* SBT2055, B cells could produce more IgA in Peyer's patch and small intestines of mice [65]. Through oral administration of VSL#3 (a mixture of multiple probiotics) to macaques, Manuzak et al. discovered that B cells could secrete more IgA in the colon and LN [66].

### 4.4. Dendritic Cells

DC is a type of professional antigen-presenting cell (APC) that can efficiently ingest, process, and present antigens. DC eventually presents antigens to T cells to affect the differentiation of T cells. Negative immune regulation can be performed when probiotics that affect DC present antigens or differentiate to DCreg.

#### 4.4.1. Anti-inflammatory Effects

Mariman et al. showed that DC secreted high levels of IL-12p70, IL-23, and IL-10 after VSL#3 stimulated mouse bone marrow DC (BMDC) [67]. Moreover, the activation of TLR-2 receptors in DC caused the polarization of Th0 cells into Treg and high levels of IL-10 and TGF-*β* secretion in MLN after coculturing BMDC with probiotics [68]. Further evidence suggested that coculturing human peripheral blood mononuclear cells (PBMCs) with *Lactobacillus crispatus (L. crispatus)* SJ-3C-US increased the maturation of DC, the number of Treg, and the secretion of IL-10 [69].

#### 4.4.2. Antiallergic Effects

In in vivo experiments, after feeding *B. infantis* to mice allergic to tropomyosin, Fu et al. also found that the maturation of DC and number of CD103+ DCreg cells increased, which promoted the expression of IL-10, TGF-*β*, and Foxp3 mRNA in Treg [70]. Some studies have shown that feeding *L. paracasei* L9 reduced the maturation of DC and increased the expression of CD103 and number of Treg in the MLN, Peyer's patch, and spleen of mice allergic to *β*-lactoglobulin [71]. In their in vitro experiments, Adam et al. extracted BMDC from mice allergic to house dust mites and then cocultured BMDC with the *Escherichia coli* Nissle 1917 strain. They found that activation of the TLR-4 pathway could promote DC differentiation. Additionally, activation of NF-*κ*B and mitogen-activated protein kinase (MAPK) pathways can promote DC to secret more IL-10 and IL-12 [72]. Some studies have also shown that after *E. coli* O83:K24:H31 stimulating DC from cord blood of pregnant women (CBDC), the differentiation and maturation of CBDC increased. In addition, CBDC expressed a higher level of CD83 and secreted more IL-10 [73].

#### 4.4.3. Other Aspects of Negative Immune Regulation

By coculturing human PBMCs with *Lactobacillus reuteri (L. reuteri)* DSM 17938, Haileselassie et al. found that the expression of CCR7 increased in DC. Moreover, the expression of Foxp3 and IL-10 in Treg also increased [74]. In addition, research showed that after *Kluyveromyces marxianus* (a fungus that provides beneficial effects like probiotics) stimulated PBMCs, they found DC secreted more IL-12, IL-1, IL-6, and IL-10, which promoted the polarization of naive T cells to Treg [75].

### 4.5. Natural Killer Cells

NK cells are involved in antitumor, antiviral, hypersensitivity, and immunoregulation activities. When probiotics promote the production of NK cells, they can positively regulate immunity. Conversely, when probiotics inhibit the number of NK cells, they negatively regulate immunity. Johansson et al. stimulated PBMCs with *Staphylococcus aureus* (*S. aureus*) and then cocultured the cells with *L. rhamnosus* GG and *L. reuteri* DSM 17938. They found that *S. aureus*-induced T cells and NK cells to proliferate and produce IFN-*γ*, but probiotics *L. rhamnosus* GG and *L. reuteri* DSM 17938 inhibited this effect [76]. In the study by Gong et al., the cytotoxicity of NK cells was enhanced after feeding mice *Bacillus subtilis (B. subtilis)* BS02 and BS04, and there were changes to CD4+ and CD8+ T cells as well as the level of IFN-*γ* [77]. Studies have shown that high expression of cytotoxic receptors and IL-22 in NK cells can be achieved by NK-92MIX cell coculturing with *L. plantarum* [78]. After oral administration of *Bifidobacterium longum (B. longum)* MM-2 to mice given an intranasal flu virus, Kawahara et al. found that the amounts of IL-6 and TNF-*α* in BALF were reduced. Additionally, the activity of NK cells in the lungs and spleen was elevated [79]. Some researchers used AJ2 (a mixture of 8 probiotics) to stimulate PBMCs and found that NK cells were activated, and the release of inflammatory cytokines was reduced [80].

### 4.6. Other Adaptive Immune Cells

In addition to acting on T cell and B cells, probiotics can also work on other adaptive immune cells, including follicular helper T cells (Tfh) and *γδ* T cells. The main function of Tfh is to assist B cells in participating in humoral immunity. The main function of *γδ* T cells is in innate immunity, as they can both recognize cancer antigens and kill cancer cells. Scharek-Tedin et al. fed *Bacillus cereus (B. cereus)* var. toyoi to weaned piglets. As a result, they found *γδ* T cells were significantly reduced in the blood [81]. Arai et al. showed that feeding heat-killed *L. paracasei* MCC1849 could increase the number of Tfh in Peyer's patch of mice [82].

### 4.7. Other Innate Immune Cells

Other innate immune cells, including macrophages, neutrophils, and mast cells, could also be influenced by probiotics. Macrophages are a type of phagocytic cell whose main function is to phagocytose pathogens and activate immune cells to respond to pathogens. Neutrophils can perform chemotaxis, phagocytosis, and bactericidal actions and defense. Mast cells can secrete a variety of cytokines and participate in immune regulation. Mast cells can also release allergic mediators to mediate allergic reactions. Through in vivo experiments, Juan et al. showed that after feeding *C. butyricum* CGMCC0313-1 to OVA-allergic mice, the degranulation of mucosal mast cells was inhibited and the infiltration of lung inflammatory cells was also reduced. In BALF, MMP-9 was reduced and IL-10 was increased [83]. In addition, Kim et al. fed *L. acidophilus* to colitis mice and found that M2 macrophages increased in the peritoneal cavity [49]. Through in vitro experiments, some researchers found that coculturing mice bone marrow-derived neutrophils with *L. rhamnosus* GG could inhibit the phagocytic ability and the cytotoxicity of neutrophils [84]. Carasi et al. cocultured human PBMCs with *Enterococcus durans (E. durans)* (EP-1) and found that IL-6 secretion was significantly reduced, while IL-10 secretion increased. After feeding mice EP-1, they found that the expression levels of IL-17, IL-6, IL-1, IFN-*γ*, and CXCL1 were remarkably reduced in Peyer's patch [85]. Studies by Gong et al. showed that feeding mice with *B. subtilis* BS02 and BS04 could enhance the phagocytosis of monocytes in mice [77].

### 4.8. Immunomodulatory Effects of Probiotic Fungi

In addition to probiotic bacteria, some fungi also have immunomodulatory effects, which can improve the host microecological balance and regulate the host immune system. Smith et al. cultured *K. marxianus* and *S. boulardii* with DCs, respectively, they found that DCs secreted increased levels of IL-12, IL-1*β*, IL-6, and IL-10. Besides, they found that the use of these two fungi cell wall extracts, *β*-glucan, could stimulate DC receptor Dectin-1, allowing DCs to secrete IL-1*β*, IL-6, and IL-10, but not including IL-12. Finally, they cultured *K. marxianus* and *S. boulardii* with the DC-naive T cell cocultured system; they found that *K. marxianus* induced the differentiation of naive T cells to Foxp3+ Treg, increased secretion of IL-10, and controlled inflammation. Moreover, *S. boulardii* could induce differentiation of naive T cells to Th1, resulting in an increased secretion of IFN-*γ* [75]. Thomas et al. cocultured bone marrow-derived DCs from Crohn's disease (CD) and ulcerative colitis (UC) patients with *S. boulardii*; they found that DCs secreted less TNF-*α* but more IL-6 and IL-8 [86]. Interestingly, the same research team cocultured *S. boulardii* with DCs isolated from PBMCs; they found that DCs secreted less TNF-*α* and IL-6 but more IL-10, thereby inhibiting T cell proliferation [87]. By coculturing DCs with *S. boulardii* and *K. marxianus* CBS1553, respectively, Smith et al. found that both *S. boulardii* and *K. marxianus* CBS1553 can promote IL-12, IL-10, IL-6, TNF-*α*, and IL-1*β* secretion [88]. In addition, by, respectively, coculturing mouse bone marrow-derived DCs and spleen cells with *β*-glucan extracted from the cell wall of *Saccharomyces cerevisiae* (*S. cerevisiae*), Karumuthil-Melethil et al. found that DCs and spleen cells could secrete increased IL-10, TGF-*β*1, and IL-2 [89].

Xu et al. firstly stimulated mouse macrophages with LPS, and then added *S. cerevisiae*, and found that *S. cerevisiae* inhibited the production of IL-1*α*, IL-1ra, and IL-27 by macrophages, of which mechanism may be related to the inactivation of the mitogen-activated protein kinase and TLR2 pathway in macrophages [90]. In addition, by feeding *S. cerevisiae* IFST062013 to mice, Fakruddin et al. found that high doses of *S. cerevisiae* IFST062013 increased the expression of TLR-2 and IFN-*γ* genes in the intestinal mucosa of mice, while Foxp3, TGF-*β*, and IL-4 gene expression decreased. They also found an increase in IL-10 in mouse serum [91]. Maccaferri et al. cocultured *K. marxianus* B0399 with PBMCs and found more IL-1*β*, IL-6, MIP-1*α*, and TNF-*α* released. In another experiment, they used LPS to stimulate PBMCs with *K. marxianus* B0399 and found the ability of LPS to trigger an inflammatory response was attenuated by *K. marxianus* B0399. Besides, *K. marxianus* B0399 can significantly reduce the concentration of proinflammatory cytokines TNF-*α*, IL-6, and MIP-1*α* secreted by PBMCs; however, IL-1*β* was increased [92]. By giving oral administration of *Scytalidium acidophilum* (*S. acidophilum*) to broilers chickens, Huang et al. found an increase in serum IgA [93]. Interestingly, after mice were infected with *C. difficile*, the mice that continued to be infected with *Candida albicans* (*C. albicans*) expressed higher levels of IL-17A in infected tissues than the mice that were not continued to be infected with *C. albicans*. This improves the survival rate after *C. difficile* infection. *C. albicans* may be a potential probiotic [94].

By feeding mice with Tibetan mushroom (a drink which was produced by fermentation of more than a dozen bacteria and yeasts), Diniz et al. found that the granuloma induced by cotton balls was significantly inhibited. Meanwhile, using carrageenan, dextran, and histamine to stimulate rats to get paw edema was also significantly reduced. But the experiment did not show which kind of bacteria or fungi was responsible for the anti-inflammatory function [95]. Zhang et al. fed mice which were allergic to peanut with ImmuBalance (a fermented soy product from *Aspergillus* and lactic acid); the results showed histamine and IgE levels were decreased in mice sera. Additionally, the amount of IL-4, IL-5, and IL-13 in mouse spleen cells was significantly reduced [96].

## 5. Efficacy and Safety of Probiotics and FMT in Clinical Trials and Application

### 5.1. Efficacy and Safety of Probiotics

To be effective at their likely sites of action, probiotics need to be able to survive stomach acid, bile, and digestive enzymes and to be viable for the duration of their shelf lives. Many products (e.g., yogurt) on supermarket shelves do not meet even these most basic standards [97]. To date, clinical trials have not been performed to test whether probiotics taken orally lose their efficacy over time. Additionally, probiotics are generally regarded as safe, but there may still be risks in certain disease populations [98]. To ensure patient safety, the participating patients were provided information both orally and in writing and were instructed to follow all instructions and attend clinical follow-ups with their usual gastroenterologist.

As described above, some probiotics have been shown to have anti-inflammatory effects and promote maintenance of the gut intestinal barrier in vitro and in murine models of IBD. This outcome may give credence to their use as a treatment option in human IBD. The results of clinical trials have been mixed, with some studies showing an improvement in the maintenance of remission or induction of remission with probiotics, while other trials have failed to show any benefit (summarized in Table 2). In a randomized controlled trial (RCT) designed by Krag et al., supplementation with profermin (contained *L. plantarum* 299v) was found to be safe and well-tolerated and to definitely reduce the simple clinical colitis activity index (SCCAI) scores at a statistically and clinically significant level in patients with mild-to-moderate ulcerative colitis (UC) with a flare-up [99]. Fedorak et al. found early treatment (at day 90 after ileocolonic resection and reanastomosis) with VSL#3 had a larger impact on the prevention of Crohn's disease (CD) recurrence than late treatment (from days 90 to 365) [100]. Yoshimatsu et al. conducted a single-center RCT and found that probiotic (a bio-three tablet, containing *Streptococcus faecalis* (*S. faecalis*) T-110, *C. butyricum* TO-A, and *Bacillus mesentericus* (*B. mesentericus*) TO-A) therapy was useful for preventing relapses of inactive UC in patients who were already in remission [101]. In a multiple-center study, Tamaki et al. found that supplementation with *B. longum* 536 (BB536) was well-tolerated and reduced the UC disease activity index (UCDAI) scores, Rachmilewitz endoscopic index (EI), and Mayo subscores after 8 weeks in Japanese patients with mild to moderately active UC [102]. By contrast, Ahmed et al. designed a prospective randomized crossover study. They found that there was no difference in the colonic microflora between patients with CD or UC and that the spectrum of the gut microflora was not altered by oral synbiotic administration, which contained 4 strains of probiotics, *L. acidophilus* LA-5, *L. delbrueckii* subsp. bulgaricus LBY-27, *B. animalis* subsp. lactis BB-12, and *S. thermophilus* STY-31 [103]. In another prospective study, Bourreille et al. showed that although the probiotic yeast *S. boulardii* was safe and well tolerated, it did not appear to have any beneficial effects for patients with CD in remission after steroid or salicylate therapies [104]. Some clinical trials also proved that probiotics could not be used as the main treatment method for IBD. Petersen et al. used probiotics *E. coli* Nissle together to treat acute UC after the antibiotic ciprofloxacin. They found that there was no benefit in the use of *E. coli* Nissle as an add-on treatment to conventional therapies for active UC. Furthermore, treatment with *E. coli* Nissle without a previous antibiotic cure resulted in fewer patients reaching clinical remission [105]. Recently, a meta-analysis showed VSL#3 could be effective for inducing remission in active UC. Probiotics may be as effective as 5-aminosalicylates (5-ASAs) in preventing relapse of quiescent UC. The efficacy of probiotics in CD remains uncertain, and more evidence from RCTs is required before their utility is known [106].

In clinical trials of other inflammatory and immune diseases, probiotics also showed an immunomodulatory effect. Sindhu et al. provided 124 children with gastroenteritis *L. rhamnosus* GG (LGG) ATCC 53103 or placebo, and they found that LGG had a positive immunomodulatory effect for improving intestinal function in children with rotavirus and cryptosporidial gastroenteritis [107]. Maldonado-Lobón et al. carried out a 3-year study to show that early administration of the probiotic of *Lactobacillus fermentum* (*L. fermentum*) CECT5716 in an infant formula was safe, and differences were observed on the incidence of infectious and noninfectious diseases or disorders related to intestinal function [108]. In recurrent aphthous stomatitis (RAS), Mimura et al. found that a symbiotic treatment based on a fructooligosaccharide, *Lactobacillus*, and *Bifidobacterium* composition produced an alteration in the Th2 serological immune profile in the direction of Th1 and improved pain symptomatology [109]. Savino et al. used *L. reuteri* to treat patients suffering from infantile colic, and they found that infants with colic treated with *L. reuteri* for 30 days had significantly increased forkhead box P3 (FOXP3) expression, which could produce more Treg and, ultimately, reduced fecal calprotectin [110]. Dennis-Wall et al. determined whether consuming *L. gasseri* KS-13, *B. bifidum* G9-1, and *B. longum* MM-2 would improve quality of life during allergy season by increasing the percentage of Tregs and inducing tolerance [111]. Kim et al. identified a population of atopic dermatitis (AD) patients with a good clinical response to probiotic treatment. All patients were given *L. plantarum* CJLP133 once a day for 12 weeks. Their results suggested that a subgroup of patients with a specific AD phenotype showing an immunologically active state (high total IgE, increased expression of TGF-*β*, and high numbers of Treg) might benefit from probiotic treatment [112]. Sheikhi et al. also investigated immune state changes with probiotics in AD. They found that *L. delbrueckii* subsp. bulgaricus could modulate the secretion of Th1- and Th2-Treg-related cytokines in AD patients [113]. In addition to Th1- and Th2-Treg-related cytokines, Rø et al. found that perinatal maternal probiotic supplementation with a combination of LGG, *B. animalis* subsp. lactis Bb-12 (Bb-12), and *L. acidophilus* La-5 (La-5) reduced the proportion of Th22 cells in 3-month-old children with AD [114]. Another study showed that only probiotics had an effect on Th17, but no effect on the relative frequencies of Th1, Th2, and Treg cells among circulating PBMCs; on plasma cytokine levels; and on in vitro production of cytokines by T cells [115]. In addition to T cells, probiotics also could affect NK cells. Lee et al. found that daily consumption of dairy yogurt containing *L. paracasei* ssp. paracasei, B. lactis, and heat-treated L. plantarum could be an effective option to improve immune function by enhancing NK cell function and IFN-*γ* concentration [116]. In enthesitis-related-arthritis category of juvenile idiopathic arthritis (JIA-ERA), probiotic VSL3# capsules were well-tolerated but failed to show any significant immune (frequencies of Th1, Th2, Th17, and Treg cells in blood, serum cytokines IFN-*γ*, IL-4, IL-17, IL-10, TNF-*α*, and IL-6) or clinical effects [117]. Another study investigating immune responses among sedentary young males showed the total leukocytes, total lymphocytes, T lymphocytes, T-helper, T-cytotoxic, B lymphocytes, and NK cell counts in peripheral blood were not significantly affected by the probiotics [118]. Komano et al. found that heat-killed *Lactococcus lactis* (*L. lactis*) JCM 5805 (LC-Plasma) supplementation relieved morbidity and symptoms of URTI via activation of plasmacytoid DC (pDC) and decreased fatigue accumulation during consecutive high-intensity exercise in athletes [119].

As described above, the effects of probiotic treatment in human studies are often variable, and there are inconsistencies between different clinical trials, undoubtedly related to the fact that different multistrain probiotic combinations have been used in variable dose frequencies. It is therefore difficult to draw clinically relevant conclusions about the effects of probiotics in human studies.

### 5.2. Efficacy and Safety of FMT

FMT is a complex intervention that involves multiple components, ranging from donor selection to the methods of transplantation (for example, colonoscopy) and several organizational levels, such as the use of stool banks or analysis of gut microbiota composition by a biologist [120]. The factors that could affect the efficacy and safety of FMT are unknown. In addition, multiple components of FMT (such as donor screening, methods for collecting stool, preparation, and transplantation) could differ among studies [121]. In IBD clinical trials (Table 2), Jacob et al. carried out a single FMT delivery by colonoscopy for active UC using a 2-donor fecal microbiota preparation. Mucosal CD4+ T-cell analysis revealed a reduction in both Th1 and Treg post-FMT [122]. Goyal et al. found that a single FMT was relatively safe and could result in a short-term response in young patients with active IBD. Responders possessed increased fusobacterium prior to FMT and demonstrated more significant microbiome changes compared to nonresponders after FMT [123]. Karolewska-Bochenek and colleagues also proved that FMT had beneficial effects on pediatric UC and CD colitis, and FMT was well-tolerated and safe. However, they emphasized that a proper protocol of FMT administration was crucial for treatment efficacy [124]. In the same year, Pai and Popov summarized an optimal and detailed multiple-center RCT protocol of FMT for pediatric IBD [125]. For CDI patients with IBD, Meighani et al. revealed that FMT could provide a good alternative treatment option with high success rates for recurrent or refractory CDI in patients with well-controlled IBD who fail standard antimicrobial therapy [126]. Khanna et al. showed that CDI patients with IBD had a higher proportion of the original community after FMT and lacked improvement of their IBD symptoms and increased episodes of CDI in a long-term follow-up [127]. Another pilot study suggested that the microbial imbalances in CDI + UC recipients more closely resemble those of the CDI-only recipients compared to the UC-only recipients after a single FMT [128].

In an age of reductionist science and targeted therapeutic interventions, FMT seems oddly unsophisticated. However, FMT has been shown to be a highly efficacious, safe, and cost-effective therapy for immune diseases, especially IBD.

## 6. Conclusions and Future Perspectives

Probiotics have a large spectrum and have been used in main diseases, such as IBD, necrotizing enterocolitis (NEC), irritable bowel syndrome IBS, diarrhea, and other gastrointestinal diseases, in vivo and in vitro. Due to their ability to regulate systemic immune function, probiotics have recently attracted attention in the development of new treatments for obesity, insulin resistance syndrome, type 2 diabetes mellitus and nonalcoholic liver steatosis, hepatic encephalopathy, autism and chronic kidney disease, allergic asthma, atopic dermatitis (AD), acne, rheumatoid arthritis, prevention of dental caries, preventive treatment of an infection, and other fields. In addition, the use of probiotic strains as carriers of antigen delivery is a viable alternative strategy to overcome the shortcomings of vaccines. However, despite their active role in various tumor diseases, probiotics also have side effects associated with anticancer therapies.

The immunomodulation induced by probiotics is a complex interaction between different hosts and microorganisms, so the immunomodulatory characteristics of specific probiotics cannot be generalized. Presently, the composition, dosage, course of treatment, specific mechanism of action, and efficacy of probiotics used in clinical treatment have not been standardized. Overall, probiotics are generally considered safe, but there is growing evidence of widespread concern about the safety of probiotics. In 2002, a joint report by the World Health Organization and the United Nations Food and Agriculture Organization showed that probiotics can cause four side effects, namely, systemic infection, harmful metabolic activity, excessive immune stimulation, and gene transfer in susceptible individuals. Recently, two reports in September 2018 also noted the unknown aspects of the safety of probiotics at this stage and raised concerns in the scientific community about studying adverse reactions to probiotics. Zmora et al. [129] emphasize that the colonization of probiotics is highly personalized and that different individuals have different sensitivity to different probiotic colonization. The host microbiome influences probiotic colonization through competitive rejection of the same species and specific immune mechanisms. The intake of probiotics did not significantly affect the composition of the symbiotic community but instead stimulated the response of the host immune system. Therefore, we suggest that it is necessary to develop personalized probiotics from the perspective of the specificity of the intestinal flora and host physiology. When a clinical application of probiotics is selected, it should gradually transform from empirical treatment to evidence-based treatment, and suitable individualized treatment plans should be developed for patients using evidence-based treatments.

Suez et al. [130] reported that in mice and mixed probiotic intervention in healthy subjects and fecal bacteria autograft (aFMT) of antibiotics might improve the recovery of the intestinal flora after disturbance; the study illustrated that compared with spontaneous recovery, probiotic preparations significantly delayed the host's feces and the reconstruction of the intestinal mucosa flora and host the transcriptome of recovery. Moreover, this study showed that it is difficult to be fully recovered; this is mainly because of the soluble factors that secreted probiotic bacteria inhibition, and probiotics in the potential beneficial effects of antibiotic therapy possibly will be offset by intestinal mucosa recovery effect. It is important to note that microbiome transplantation enables rapid and nearly complete recovery of host-microbiome and transcriptome within a few days. This suggests that, compared with probiotics or prebiotics, fecal bacteria transplantation as the most direct method of intestinal flora intervention may be more effective and feasible.

Since 2013, when it was included in the FDA's official treatment guidelines for relapsing *C. difficile*, fecal transplants have seen more comprehensive development worldwide. Compared to the standard use of probiotics, FMT can be explored faster and further in this area. At present, the standardization of donor screening, microflora separation and preparation, transplantation, and other aspects involved in the FMT process has begun to take shape. Recently, a large number of studies [131–135] have proposed the step-up treatment strategy of FMT: when the single FMT and multiple FMTs (greater than or equal to 2) are not effective, FMT can be combined with conventional drug therapy (such as glucocorticoid, cyclosporine, anti-TNF-beta antibody, and whole intestinal nutrition). The efficacy of each step can be improved in the next step. This FMT stepwise treatment strategy is suitable for refractory IBD, immune-related diseases [135], and severe or complex CDI [131], especially for patients who are not responsive to conventional therapeutic drugs. At the same time, severe adverse events caused by FMT can be caused by infectious microorganisms in donor feces, which is because many infectious diseases in the donor are still difficult to be excluded. Therefore, FMT-related adverse events in specific populations should be prevented, especially those with low immunity. During FMT treatment through the digestive tract, improper fecal bacteria infusion technology and process may also lead to nausea, vomiting, aspiration, and other adverse events. In order to prevent FMT transmission diseases, strict donor screening should be carried out, and FMT treatment decisions, methods, short-term and long-term follow-up safety evaluation, and supervision will be the focus of future research.

To sum up, personalized probiotics intervention and standardized fecal bacteria transplantation should be challenges and prospects for future research on the intervention model of intestinal flora. Furthermore, increasing evidence shows that the microbiome has potential effects outside the intestinal tract, such as vagina and sinus tract [136], urethra, [137] and skin [138]. Therefore, future research should focus on a specific use of microbiome in different organs.

## Figures and Tables

**Table 1:** The immunomodulatory components of Lactobacillus and Bifidobacterium.

Probiotic genera	Probiotic strains	Immunomodulatory components of probiotics	References
*Lactobacillus*	*L. acidophilus*, *L. amylovorus*, *L. bulgaricus*, *L. crispatus*, *L. casei*, *L. gasseri*, *L. helveticus*, *L. johnsonii*, *L. pentosus*, *L. reuteri*, *L. paracasei*, *L. plantarum*, *L. rhamnosus*	(1) Lipoteichoic acid stimulates NO synthase (2) Lipoproteins and LTA can potentially signal through binding to TLR2 in combination with TLR6 (3) Unmethylated DNA fragments containing CpG motifs mediate anti-inflammatory effects via TLR9 signaling at the epithelial surface (4) Highly O-acetylated peptidoglycan might affect the release of NLR stimulating PG fragments and innate immune responses of antigen-presenting cells such as dendritic cells and macrophages (5) EPS and other cell wall polysaccharides could be recognized by CLRs that are involved in the recognition and capture of antigens by antigen-presenting cells such as dendritic cells and macrophages	[139–143]

*Bifidobacterium*	*B. animalis*, *B. breve*, *B. infantis*, *B. bifidum*, *B. lactis*, *B. catenulatum*, *B. longum*, *B. adolescentis*	(1) Lipoteichoic acid stimulates NO synthase (2) Bifidobacterial proteins are one of the targets of human immunoglobulins, notably IgA (3) Although no specific host receptors have been found, EPS has been recognized as an effector of the interaction between probiotics and the host immune system (4) *Bifidobacteria* possess genomes with high G+C proportions, and unmethylated CpG motifs derived from them can interact with the TLR 9 present on immune cells (5) The peptidoglycan hydrolase TgaA is shown to induce IL-2 production in the monocyte-derived dendritic cell, the key cytokine in Treg cell expansion (6) The specific interaction between pili and gastrointestinal mucosa	[41, 144–146]

NO: nitric oxide; PG: peptidoglycan; LTA: lipoteichoic acid; LPS: lipopolysaccharide; EPS: exopolysaccharides.

**Table 2:** RCT clinical trials of probiotics and FMT treatment in IBD.

Researcher/country	Year	Single/multiple-center study	Strains of probiotics/delivery way of FMT	No. of enrolled patients	Diseases	Period of observation	Efficacy	Safety
Ahmed/UK [103]	2013	Single	*Lactobacillus acidophilus* LA-5, *Lactobacillus delbrueckii* subsp. *bulgaricus* LBY-27, *Bifidobacterium animalis subsp. lactis* BB-12, and *Streptococcus thermophilus* STY-31	20	CD and UC	2 months	No	Not mentioned
Krag/Denmark [99]	2013	Single	*Lactobacillus plantarum* 299v	74	UC	2 years	Yes	Yes
Bourreille/France [104]	2013	Single	*Saccharomyces boulardii*	165	CD	52 weeks	No	Yes
Petersen/Denmark [105]	2014	Single	*Escherichia coli Nissle*	100	UC	7 weeks	No	Not mentioned
Fedorak/Canada [100]	2015	Multiple	4 strains of *Lactobacillus*, 3 strains of *Bifidobacterium*, and 1 strain of *Streptococcus salivarius subspecies thermophilus*	119	CD	1 year	Yes	Yes
Yoshimatsu/Japan [101]	2015	Single	*Streptococcus faecalis* T-110, *Clostridium butyricum* TO-A, and *Bacillus mesentericus* TO-A	46	UC	1 year	Yes	Yes
Tamaki/Japan [102]	2016	Single	*Bifidobacterium longum* 536	56	UC	8 weeks	Yes	Yes
Jacob/USA [122]	2017	Single	Colonoscopy	20	UC	4 weeks	Yes	Yes
Meighani/USA [126]	2017	Single	Colonoscopy	201	CD and UC	2 years	Yes	Not mentioned
Karolewska-Bochenek/Poland [124]	2017	Single	Nasoduodenal tube or gastroscopy	10	CD and UC	2 weeks	Yes	Yes
Goyal/USA [123]	2018	Single	Upper and lower endoscopy	21	CD, UC, and IC	6 months	Yes	Yes
Mintz/USA [128]	2018	Single	Colonoscopy	26	UC	3 months	Yes	Not mentioned
